# Prevalence of *TERT* Promoter Mutations in Orbital Solitary Fibrous Tumors

**DOI:** 10.3390/cimb46020095

**Published:** 2024-02-10

**Authors:** David Sinan Koca, Vladimir Kolpakov, Jana Ihlow, Maximilian von Laffert, Katharina Erb-Eigner, Hermann Herbst, Karen Kriese, Leonille Schweizer, Eckart Bertelmann

**Affiliations:** 1 Department of Ophthalmology, Charité—Universitätsmedizin Berlin, Campus Virchow-Klinikum, Augustenburger Platz 1, 13353 Berlin, Germanyeckart.bertelmann@charite.de (E.B.); 2 Institute of Pathology, Charité—Universitätsmedizin Berlin, Corporate Member of Freie Universität Berlin and Humboldt—Universität zu Berlin, Charitéplatz 1, 10117 Berlin, Germany; 3 Berlin Institute of Health Charité Clinician Scientist Program, Berlin Institute of Health at Charité—Universitätsmedizin Berlin, Berlin Institute of Health Biomedical Innovation Academy, Anna-Louisa-Karsch-Str., 210178 Berlin, Germany; 4 Department of Diagnostics, Institute of Pathology, Universitätsklinikum Leipzig AöR, Liebigstraße 26, 04103 Leipzig, Germany; 5 Department of Radiology, Charité—Universitätsmedizin Berlin, Campus Virchow-Klinikum, Augustenburger Platz 1, 13353 Berlin, Germany; 6 Department of Pathology, Vivantes Hospital Neukölln, Vivantes Netzwerk für Gesundheit GmbH Berlin, Rudower Straße 48, 12351 Berlin, Germany; 7 Department of Neuropathology, Charité—Universitätsmedizin Berlin, Corporate Member of Freie Universität Berlin and Humboldt—Universität zu Berlin, Charitéplatz 1, 10117 Berlin, Germany; 8 German Cancer Consortium (DKTK), Partner Site Berlin, German Cancer Research Center (DKFZ), 69120 Heidelberg, Germany; 9 Edinger Institute, Institute of Neurology, University of Frankfurt am Main, 60528 Frankfurt am Main, Germany; 10 German Cancer Consortium (DKTK), Partner Site Frankfurt–Mainz, German Cancer Research Center (DKFZ), 69120 Heidelberg, Germany; 11 Frankfurt Cancer Institute (FCI), 60596 Frankfurt am Main, Germany

**Keywords:** orbital solitary fibrous tumor, *TERT* promoter mutation, NAB2-STAT6, diffusion-weighted imaging (DWI), signal intensity void, chemical shift artifact

## Abstract

The orbital manifestation of a solitary fibrous tumor (SFT) is exceptionally rare and poses specific challenges in diagnosis and treatment. Its rather exceptional behavior among all SFTs comprises a high tendency towards local recurrence, but it rarely culminates in metastatic disease. This raises the question of prognostic factors in orbital SFTs (oSFTs). Telomerase reverse transcriptase (*TERT*)-promoter mutations have previously been linked to an unfavorable prognosis in SFTs of other locations. We analyzed the prevalence of *TERT* promoter mutations of SFTs in the orbital compartment. We performed a retrospective, descriptive clinico-histopathological analysis of nine cases of oSFTs between the years of 2017 and 2021. A *TERT* promoter mutation was present in one case, which was classified with intermediate metastatic risk. Local recurrence or progress occurred in six cases after primary resection; no distant metastases were reported. Multimodal imaging repeatedly showed particular morphologic patterns, including tubular vascular structures and ADC reduction. The prevalence of the *TERT* promoter mutation in oSFT was 11%, which is similar to the prevalence of extra-meningeal SFTs of the head and neck and lower than that in other extra-meningeal compartments. In the present study, the *TERT* promoter mutation in oSFT manifested in a case with an unfavorable prognosis, comprising aggressive local tumor growth, local recurrence, and eye loss.

## 1. Introduction

A solitary fibrous tumor (SFT) is a mesenchymal fibroblastic neoplasm with a clinically heterogeneous appearance [1]. This can implicate a locally destructive growth pattern and malignant transformation [1,2,3]. SFTs have a ubiquitous appearance, but are categorized into extra-meningeal (pleural and extra-pleural) and meningeal SFTs owing to differences in histopathology and prognosis [4,5]. The hallmark sign of the tumor is the NAB2::STAT6 fusion oncogene (NAB2: NGFI-A-binding protein 2; STAT6: signal transduction and activator of transcription 6) [6,7]. The juxtaposition of NAB2 and STAT6 via an inverted intra-chromosomal fusion on chromosome 12 transfers the transactivation domain (TAD) of STAT6 to NAB2 [6,7,8]. This results in a gain of function mutation, leading to the dysregulation of EGR1-dependent gene expression, e.g., Insulin-like growth factor 2 (IGF2) and associated insulin-receptor pathways, which entails cellular proliferation [9,10].

According to the fifth edition of the *WHO Classification of Tumors: Soft Tissue and Bone Tumors* from April 2020, the diagnosis of an SFT should desirably demonstrate the NAB2::STAT6 fusion gene, particularly in inconclusive cases [4]. Yet, a typical histology combined with CD34 and/or STAT6 overexpression is sufficient for diagnosis [11,12,13]. Since the initial description of a ‘hemangiopericytoma’ in 1942 by Stout and Murray, the terminology and the diagnostic standards have been revised multiple times, which makes the interpretation of former work challenging [2,14]. Only recently, in 2021, was the term ‘hemangiopericytoma’ removed from the fifth edition of the *WHO Classification of Tumors: Central Nervous System Tumors* and the term should no longer be used to describe an SFT [4].

The orbital manifestation of a solitary fibrous tumor is extremely rare [15]. The incidence is estimated at 0.3–0.5/million per year [16]. The orbital solitary fibrous tumor (oSFT) often presents with painless ipsilateral proptosis, sometimes causing diplopia [3]. The evaluation of orbital tumors preferably comprises multimodal imaging [17]. This includes diffusion-weighted imaging (DWI) MRI sequences. In cases of osseous infiltration acomputed tomography (CT) is required [17,18,19]. Despite a difficult surgical approach, complete surgical dissection is essential, since adjuvant radiation puts visual function at risk and oSFTs are poorly responsive to chemotherapy [20,21]. The most important risk factor for recurrence is incomplete surgical excision [1,20,21,22].

In canonical conception, oSFTs are considered extra-meningeal unless there is secondary orbital infiltration of meningeal origin [4,13,23]. While malignancy in meningeal SFTs is purely assessed using histological parameters (WHO grade I–III), risk stratification in extra-meningeal SFTs also considers epidemiological parameters [16,24,25,26]. Recent evidence suggests that the underlying NAB2::STAT6 fusion variant impacts the risk of recurrence and correlates with the anatomical compartment [8,27,28]. There are more than 40 breakpoint variants, with the most common being NAB2ex4::STAT6ex2/3 and NAB2ex6::NAB6STAT6ex17 [28,29]. While NAB2ex4::STAT6ex2/3 occurs in tumors with more benign characteristics, NAB2ex6::NAB6STAT6ex17 is correlated with younger age and more aggressive behavior [27,29].

**Figure 1:**
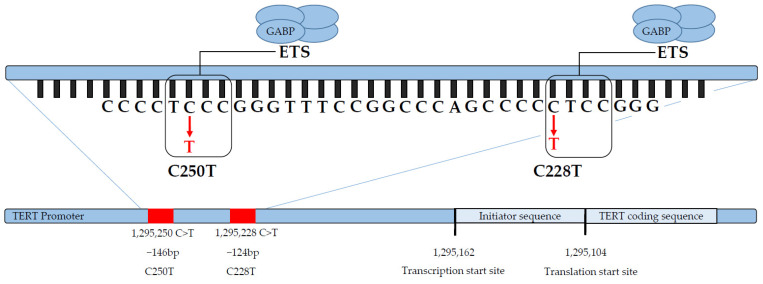
Schematic illustration of the DNA sequence of the *TERT* promoter mutation at Chromosome 5p. The *TERT* promoter hotspot mutations C228T and C250T occur at −146 bp and −124 bp from the translation start site of the *TERT* gene (ATG; reference genome hg19). C228T and C250T induce changes that create new binding sites for ETS family members, e.g., GABPs. GABPA hereby forms heterotetramers with GABPB1 or GABPB2 in order to exert a master regulatory function on the mutated *TERT* promoter.

In the research of reliable prognostic parameters, mutations of the telomerase reverse transcriptase (*TERT*) promoter have been extensively studied, both in meningeal and extra-meningeal SFTs [3,8,30,31]. Telomerase is an enzyme that counteracts chromosomal shortening [30,32]. Mutations of the *TERT*-promoter alter cellular senescence and represent an early and one of the most common mutations in a vast variety of cancers [30,32,33,34,35]. The two most common *TERT* promoter mutations are found at −124 and −146 base pairs from the *TERT* translation site (see Figure 1) [36]. These hotspot mutations are termed C228T and C250T. They are mutually exclusive and typically heterozygous [35]. Occurring in non-coding regions, these mutations create de novo binding sites for E-26 family transcription factors (ETSs), e.g., GA-binding proteins (GABPs) [30,35,37,38,39,40] (see Figure 1). In consequence, *TERT* promoter activity as well as *TERT* gene transcription is reinforced [35,36]. The frequency of *TERT* promoter mutations in meningeal SFTs (32%, 50%) seems to be higher than in extra-meningeal SFTs (24%, 26%) [8,30,31,41]. However, in extra-meningeal SFTs, they have been linked to an adverse outcome [3,8,30,31].

The aim of our study was to evaluate the prevalence of *TERT* promoter mutations of oSFTs in the context of clinical appearance, histology, and molecular pathology. To our knowledge, *TERT* promoter mutations in SFTs have never been studied in the orbital compartment exclusively [8,30,31,41].

## 2. Materials and Methods

### 2.1. Ethical Approval

This study was conducted in accordance with the Ethics Committee of Charité Campus-Virchow Klinikum (protocol code EA2/182/23 8 September 2023). This study was undertaken in accordance with all rules, directives, and guidelines for good scientific practice of the University Hospital Charité Berlin and according to the Declaration of Helsinki.

### 2.2. Study Design and Case Research

We performed a retrospective case analysis of surgically dissected oSFTs during the years 2017 to 2019 at the Department of Ophthalmology, Campus Virchow—Klinikum, at the University Hospital Charité Berlin. Cases of surgically resected oSFTs were investigated using the medical information system of the University Hospital Charité Berlin (SAP). We researched the operation procedure codes for orbitotomy (OPS 5-160.0–5-160.4) excluding the additional code of decompression (5-169), which was used for the surgical treatment of compressive endocrine orbitopathy. The remaining cases were screened on the basis of the definitive histopathological results. Of the 469 cases of orbitotomy surgery, eight cases of oSFTs were confirmed by histology. One new case was found incidentally as a patient presented with recurrent exophthalmos and then underwent tumor surgery during the research period.

### 2.3. Clinical and Radiographic Analyses

Cases were analyzed regarding demographic data (age at first diagnosis, sex), symptoms at initial presentation (laterality, proptosis/exophthalmos, swelling, vision disorders, pain), best corrected visual acuity, intraocular pressure, and ophthalmologic and systemic preconditions. The patients’ history and course of disease were reviewed, including the department of initial presentation (neurosurgery, ophthalmology, oral and maxillofacial surgery, otorhinolaryngeal medicine), surgical treatment (craniotomy, orbitotomy, local excision, enucleation, or exenteration), tumor-free survival, time to relapse, and overall survival. Analysis of radiographic findings included CT scans of the head, cranial MRI scans, and orbital MRI scans. Imaging was undertaken by the Department of Radiology of the University Hospital Charité Berlin. If available, we also analyzed images obtained by external outpatient services. All imaging data were transferred to MERLIN Diagnostic Workcenter (Phönix-PACS GmbH, Freiburg im Breisgau, Germany) and analyzed by a radiologist with advanced experience in orbital imaging.

### 2.4. Histological Analyses and Immunohistochemistry

All tissue specimens were retrieved from surgical tumor resection, whereas no specimen was acquired through excisional biopsy. For morphological assessment, 3 µm sections of each sample were stained with hematoxylin–eosin (H&E). Automated immunohistochemistry was performed using a Ventana BenchMark XT immunostainer (Ventana Medical Systems, Inc., Tucson, AZ, USA). Briefly, 3 µm FFPE tissue sections were deparaffinized, rehydrated, and subjected to heat-induced epitope retrieval and endogenous peroxidase blocking with H_2_O_2_. Then, SFT slides were stained with antibodies against CD34 (Epitomics, Burlingame, CA, USA, clone EP88, 1:50), Bcl2 (1:25, Dako, Santa Clara, CA, USA, clone 124), CK MNF116 (1:1000, Dako, clone MNF116), CD31 (1:25, Dako, clone JC/70A), Vimentin (1:5000, Dako, clone V9), STAT6 (1:1000, SantaCruz, Dallas, TX, USA, clone D-1), Ki-67 (1:50, Dako, clone MIB-1), CD99 (1:200, Dako, clone 12E7), S100 (1:10000, BioGenex, Fremont, CA, USA, clone 15E2E2), p53 (1:50, Dako, clone DO-7), and p16 (1:200, Ventana, Oro Valley, AZ, USA, clone Cintec).

### 2.5. Analyses of Molecular Pathology

DNA isolation from formalin-fixed paraffin-embedded tumor samples was performed using the Maxwell RSC FFPE Plus DNA Kit (Promega Corporation 2800 Woods Hollow Road·Madison, WI 53711-5399 USA) according to the manufacturer’s instructions. The *TERT* promoter mutations (genomic position chr5, 1,295,228 C>T hg19 coordinate and chr5, 1,295,250 C>T hg19) were analyzed using Sanger sequencing (forward primer: GGATTCGCGGGCACAGAC; reverse primer: CAGCGCTGCCTGAAACTC). Details regarding the PCR protocol and conditions are available upon request. Sequencing was performed at Eurofins Genomics, Ebersberg, Germany. NAB2::STAT6 mutation and -fusion variant analyses were performed using the RNA-Archer FusionPlex sarcoma panel (Archer–Now Part of Integrated DNA Technologies, Inc. 2425 55th Street, Boulder, CO 80301, USA) according to the manufacturer’s instructions.

## 3. Results

### 3.1. Epidemiological and Clinical Parameters

**Figure 2:**
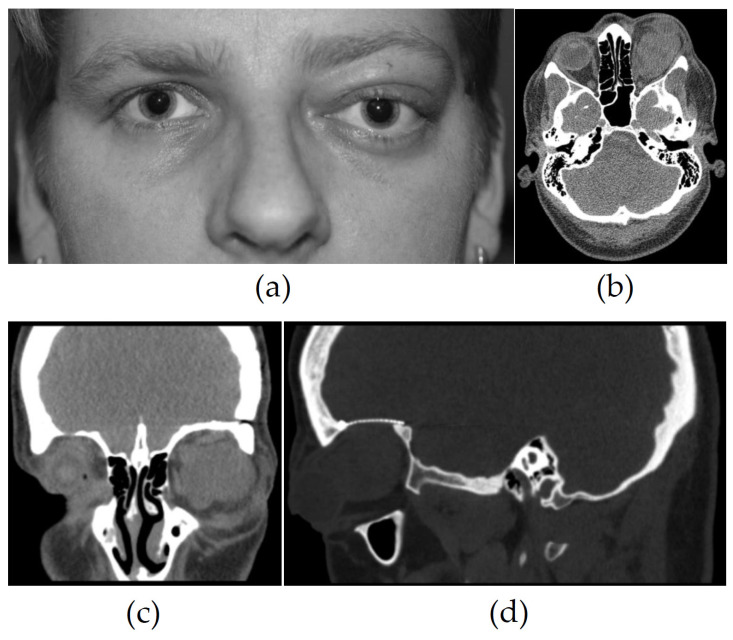
(**a**) A 33-year-old female patient with painless proptosis of the left eye and no subjective visual impairment. At first presentation, the BCVA was 0.9, IOP 21 mmHg, no history of ophthalmic disease. Computed tomography with contrast media was initially performed due to better availability. (**b**) Axial, (**c**) coronary, and (**d**) sagittal CT revealed a solid hyperdense intraconal mass with compressive growth, with no signs of osseous infiltration. After admission to our neurosurgical department, resection was performed via the transcranial pterional approach. Because of the proximity to the optic nerve, total resection could not be achieved (R1 status). After tumor reduction, the tumor did not show any relevant growth for three years and the patient was followed up regularly in our ophthalmological department. (BCVA: best corrected visual acuity.)

The study included nine patients, of which four were female and five were male. The mean age at first presentation to our hospital was 55 years (23 to 83 years, median: 46). The majority of the patients reported painless intra- or periorbital swelling (8/9) leading to ipsilateral proptosis/exophthalmos (7/9) without visual alteration (Figure 2a). In the case of altered best corrected visual acuity (BCVA), a relevant non-tumor-related ocular pathology was found. Detailed information regarding demographics and clinical parameters is presented in Table 1. One patient had a history of a periorbital tumor, which was formerly surgically removed by our ORL department. This tumor was classified as an SFT 5 years before admission to our ophthalmological department. Another patient presented with recurrent orbital mass after the R1 resection of an aggressive orbital SFT 8 years before, which was treated with adjuvant stereotactic radiation at the time. One patient had a history of a gynecologic tumor of unknown dignity, which was surgically removed with no radio- or chemotherapy being administered.

### 3.2. Imaging

**Figure 3:**
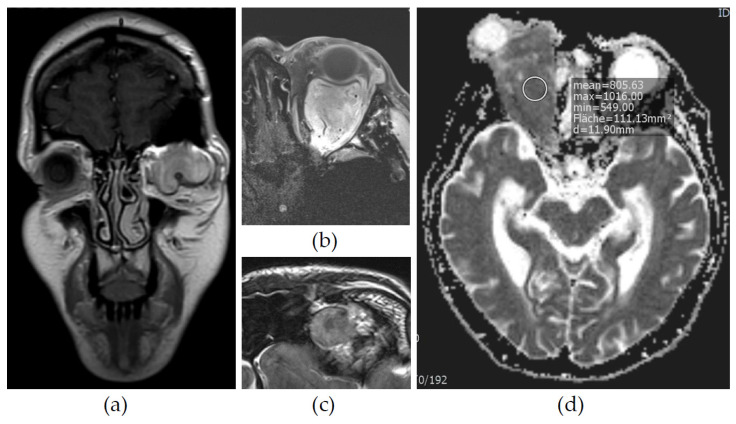
Four years later, the same patient presented with an increasing tumor growth with compressive behavior (**a**). Like the original tumor before (for CT scans, see Figure 2b–d), the recurrent tumor mass showed a signal intensity void (**b**). This phenomenon was found in 6 other tumors and is considered a radiomorphological correlate of intratumoral vessels. A chemical shift artefact was present in 3 of 9 tumors (**c**). Low ADC values (<1000 × 10^−6^ mm^2^/s) as a correlate of diffusion restriction was found in around the half of the tumors (4/9) (**d**). (**a**): 37-year-old female, contrast media T1 SE coronal MRI, arrow indicates optic nerve. (**b**): 33-year-old female, axial T1 TSE FS axial MRI, arrows indicate signal intensity void. (**c**): 27-year-old male, axial T2 TSE MRI, arrows indicate chemical shift artefact. (**d**): 87-year-old female, MRI, ADC, ROI indicates reduced mean ADC.

All patients were evaluated by cranial and/or orbital MRI. Six patients underwent computed tomography (CT). Details of the imaging are shown in Table 2. The primary manifestation was mostly intraconal (6/9) (see Figure 2b), showing a tendency of secondary extraconal growth (3/9). One tumor showed intraconal expansion after extraconal manifestation. The other tumors primarily manifested in the extraconal space (3/9). On CT, the tumors appeared hyperdense with a rather homogeneous structure (6/9) (see Figure 2b–d). MRI studies showed a homogeneous (4/9) to heterogeneous (5/9) tumor with accentuated patterns of vascularization (8/9) and mostly solid tumor growth (7/9) with the displacement of local structures (3/9) (see Figure 3a). DWI imaging showed a diffusion restriction in around half of the tumors (4/9) (see Figure 3d). In MRI scans of seven tumors, we found a signal intensity void corresponding to intratumoral vessels (Figure 3b). A chemical shift artefact was found in three tumors. As only three of the nine tumors showed this, it appeared rather inconsistently (Figure 3c). The radiographic diagnosis differed from the final histopathology, with cavernous venous malformation (formerly hemangioma) being the most important preliminary diagnosis (5/9).

### 3.3. Surgery and Follow-Up

The number of surgeries a single patient had to undergo varied from 1 surgery to 14 surgeries depending on the number of local recurrences. No event of distant metastatic disease was reported. Surgical procedures of tumor resection included eye preserving techniques (23/31) and definitive tumor resection (8/31). The eye preserving techniques included, among others, transcutaneous and local resection (7/31), lateral orbitotomy (9/31), medial orbitotomy with swinging eyelid (1/31), and pterional access with craniotomy (2/31). In seven of nine cases, eye preservation could be achieved. However, it was recommended that one patient of the seven should undergo orbital exenteration. Definitive tumor resection demanded enucleation of the eye (1/9) and exenteration of the orbit (1/9) as well as extended osseous dissection (6/32). Details of the tumor surgery and follow-up are shown in Table 3. The case with extended osseous tumor expanse needed continuous treatment by our ORL department because of insufficient local tumor control. This included hemimaxillotomy and repeated local tumor debulking surgeries. Aggressive local tumor progress made radiation therapy of the wound area necessary. As tumor progress involved the carotid canal, chemotherapy with Pazotanib + Doxorubicin was considered. The patient’s general condition did not allow chemotherapy with Anthracycline. Hence, in case of further tumor growth, the patient was planned to receive treatment with Pazotanib only. Primary tumor resection only achieved R0 resection in one case. In four cases, residual tumor mass was present (R1, 4/9) or it was unclear whether everything had been removed (Rx, 4/9). In one case, R0 resection was achieved after local recurrence (Table 4). Tissue samples for pathology were collected during every surgery. Two patients received adjuvant treatment with radiation. For another patient, radiotherapy had been recommended, but it remained unclear whether it was performed. The shortest period until relapse was 3 months due to incomplete resection. To date, the longest tumor-free interval is 198 months (Table 3). To rule out distant metastasis, tumor staging was performed in two patients. No distant metastasis was found.

### 3.4. Pathology

**Figure 4:**
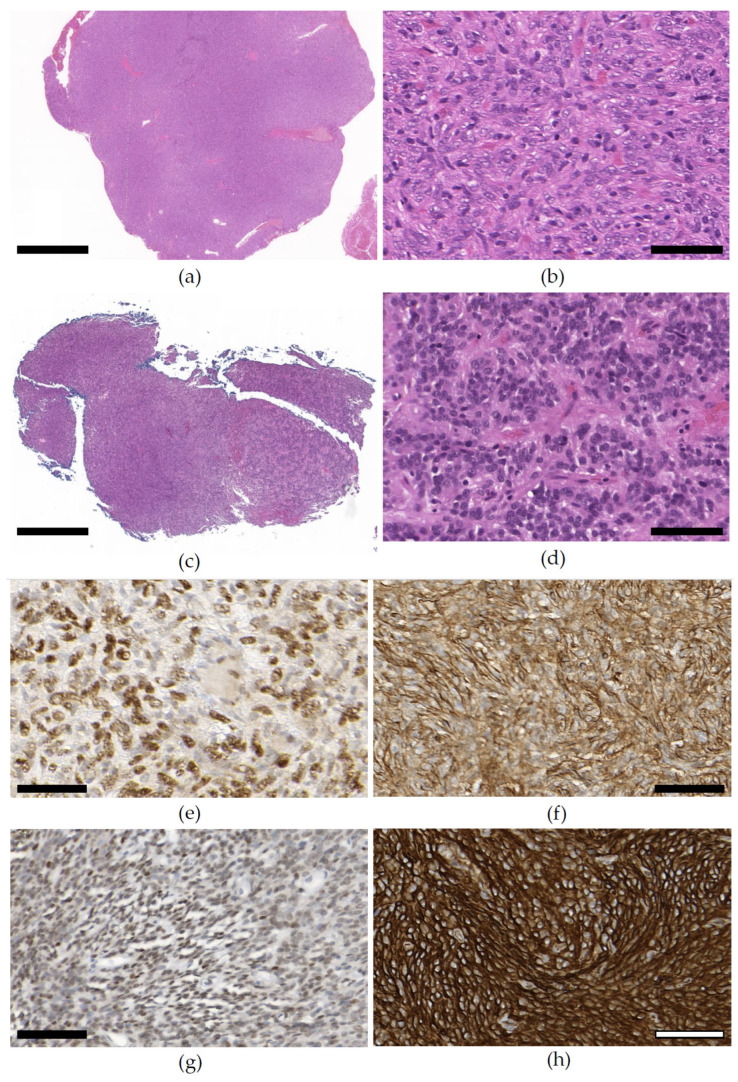
Representative pictures of histology and immunohistochemistry. The H&E staining (**a**,**b**) showed a storiform-like growth pattern with dense cellularity and a spindled-cell appearance that belonged to a tumor with less aggressive clinical behavior. The H&E staining (**c**,**d**) showed a dense cellularity with stranded bands of hyalinized collagenous tissue between the tumor cell nests that belonged to a tumor with very aggressive clinical behavior and a *TERT* promoter mutation. The diagnosis of an SFT can be given if a typical histology (**a**,**b**) and the typical immunohistochemistry with strong staining for STAT6 (**e**) and/or CD34 (**f**) is present, as was presented in the first tumor with less aggressive behavior. In contrast, the tumor with aggressive clinical behavior showed intense staining for CD34 (**h**), but only weak STAT6 positivity (**g**). (**a**,**c**): scale bar 500 µm. (**b**,**d**,**e**–**h**): scale bar 50 µm.

**Figure 5:**
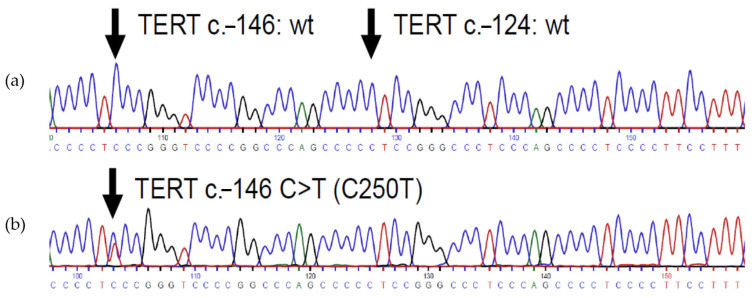
Sanger sequencing exploring *TERT* promoter mutation status of our collective: (**a**) regular Sanger sequence. (**b**) At position −146 of the base pairs, starting from the transcriptional start site, the patient has an exchange of the nucleic bases *cytosine* to thymine (C250T mutation).

Of all of the tissue samples from tumor surgery, we chose to analyze 17 representative specimens. The samples were chosen from relevant points of time, including first manifestation, local recurrence, or clinically significant progression that made organ preservation impossible and resulted in definitive surgery (e.g., enucleation or exenteration). H&E staining depicted a picture of mostly intermediate, sometimes high cellularity with a spindled cell or partially spindled cell configuration (8/9), with an epithelioid aspect in parts (3/9) (see Figure 4a–d). Signs of malignancy such as a high cellularity (3/9), a high mitotic count (>1/mm^2^) (2/9), or focal necrosis (1/9) were not common, but particularly appeared in samples from tumors with a clinically aggressive growth pattern (see Figure 4c,d) and in cases of relapse [1]. The histologic parameters are depicted in Table 4, while detailed histology information is shown in Supplementary Table S1. In immunohistochemistry (see Figure 4e–h), the tumors exhibited quite a homogeneous pattern with strong positivity for CD34, nuclear STAT-6, CD99 (see Table 5), and Bcl-2 (Supplementary Table S2), but little or no staining for CD31, CK MNF116, or S100 (Supplementary Table S2). In all cases, an NAB2::STAT6 fusion transcript was demonstrated. The most common fusion variant was NAB2ex3::STAT6ex19 (see Table 6). According to the metastatic risk prediction model from Demicco et al., most of the tumors (7/9) were of low risk, and only two of the nine showed intermediate metastatic potential [24] (see Table 4). A *TERT* promoter mutation (C250T) was present in one of the nine cases (see Figure 5). The mutation was found in all examined specimens from the patient (primary and two recurrences) and did not occur during tumor progress (see Table 6). No unaffected tissue was examined. Interestingly, the related case showed the most aggressive growth pattern by far. The patient underwent local radiation and was evaluated for systemic chemotherapy (see above).

## 4. Discussion

oSFT is an exceptionally rare occurrence with particular properties among all extra-meningeal SFTs, such as a high tendency towards local recurrence, intermediate potential of malignant transformation, and a low likelihood of metastasis [3,5]. In the present study, we described the prevalence of the *TERT* promoter mutation in SFTs in the orbit and associated pathology in nine cases.

The clinical presentation in our study cohort was very unspecific and depicted symptoms of displacement and compressive growth, as reviewed in the literature [16,18,42]. Pathognomonic paraneoplastic syndromes, like refractory hypoglycemia syndrome (Doege–Potter syndrome) or Pierre–Marie–Bamberger syndrome, did not occur. This is probably due to the tumors of the orbit being of smaller size compared to tumors in other locations, such as in the pleura or retroperitoneum [21]. Heterogeneous MRI signal intensities and CT densities have previously been linked to different densities of the soft tissue of SFTs [43]. Diffusion restriction is a strong predictor for malignancy in orbital neoplasms [17]. Meanwhile, low to intermediate ADC values could be helpful for distinguishing SFTs from benign lesions with similar morphology (e.g., cavernous venous malformation) [17]. Signal intensity void is a phenomenon arising from intra-tumoral vessels and has been shown previously in oSFTs [44]. As seven of the nine tumors depicted a signal intensity void, we considered this phenomenon to be a strong predictor for SFTs in solid and well circumscribed orbital lesions with diffusion restriction. Another radiographic clue is given by the chemical shift artefact. Chemical shift refers to the phenomenon of different precessional frequencies of protons in fat and water. This results in a shift in the spatial location of fat voxels in the frequency-encoded direction [45]. The artefact appears as a dark or bright band at the interface between water and fat (Figure 3c). Usually, chemical shift is suggestive of cavernous venous malformation [46]. Hence, in this study, cavernous venous malformation was the most frequent differential diagnosis on imaging. However, in our collective, it appeared rather inconsistently. In CNS-related locations, vascularized meningioma shows similar imaging patterns as meningeal SFTs and needs to be considered an important differential diagnosis [47,48]. Interestingly, a recent study by Liu et al. demonstrated that DWI imaging (ADC1) could differentiate between SFT and atypical meningioma with high sensitivity and specificity [49]. In conclusion, the imaging of oSFTs shows a heterogeneous tumor with varying T1/T2 intensity and low to intermediate ADC values. A signal intensity void was often present, while some oSFTs had a chemical shift artifact. Since the present study included some scans that were not performed at our clinic, signal intensities may vary.

In periorbital and orbital manifestation, curative treatment demands an aggressive local resection. The recurrence rates in our study were mainly dependent on resection status. R1 status was related to the preservation of anatomical structures that are crucial for visual function. In the majority of cases (7/9), an indistinct diagnosis at first resection explains why a conservative non-aggressive resection was performed. It has previously been reported that the surgical approach is a key factor for the recurrence rate [22]. In a study from Yang et al., tumor resection via lateral orbitotomy reached similar recurrence rates (83%) to those in the present study (88%) [20]. As the tumor dissection surgery was performed by an ‘ophthalmological’ surgical approach (local excision, swinging eyelid approach, lateral orbitotomy), the possibility of dissecting periosteum, bone, and dura was limited. According to Yang et al. [20], the ‘neurosurgical approach’, the transfronto-orbital approach, results in much lower recurrence rates (17.6%). However, the study by Yang et al. [20] evaluated the surgical resection of SFTs exclusively localized in the retrobulbar compartment with different degrees of involvement of intracranial structures and without specifying the primary site of tumor manifestation. We propose that tumor dissection should value tumor location as well as appropriate functional and anatomical status. In our study, the tumors tended to recur locally (6/9) and showed increasingly aggressive local growth.

The diagnosis of SFT demands a typical histology with CD34 and/or STAT6: spindled to ovoid cells that arrange around a branching and hyalinized system of vessels (staghorn pattern) (see Figure 4a–d) with different degrees of stromal collagen (see Figure 4b,d) [13]. Since an increased degree of dedifferentiation can lead to a loss of expression of CD34 and STAT6 (see Figure 4g), we performed an extended immunohistological panel (see Supplementary Table S2) [50]. While CD99 and Bcl-2 are a common feature of SFTs, we used other markers to exclude relevant differential diagnoses, like CD31 (e.g., vascularized malignancies like spindled-cell angiosarcoma), Cytokeratin MNF116 (e.g., epithelioid sarcoma), or S100 (e.g., malignant peripheral nerve sheath tumor) (see Supplementary Table S2) [51]. Nevertheless, we tested all tumors for the defining NAB2::STAT6 fusion oncogene and the fusion variant. NAB2::STAT6 fusion variants like NAB2ex4-STAT6ex2/3 occur in less aggressive tumors in elderly patients and are mostly localized in the pleura, while NAB2ex6-STAT16/17 correlates with a more aggressive phenotype in younger patients [29]. Georgiesh et al. showed that these differences might depend on the mutation break points in STAT6 and, therefore, looked into STAT6 integrity [27]. NAB2::STAT6 fusion variants that possess the complete STAT6 protein, namely, STAT6-ex2/3/4/5/6 (STAT6-Full), showed a better prognosis than STAT6 with only the TAD, namely, STAT6ex16/17/18/19 (STAT6-TAD). In our cohort, most of the tumors (5/8) could be considered STAT6-TAD. Although both cases where eye preservation could not be achieved were STAT6-TAD, there was no clear connection to eye preservation or local recurrence (see Table 6). In our cohort of oSFTs, *TERT* promoter mutations were less frequent (1/9) than in previous studies. However, the prevalence of *TERT* promoter mutations was never investigated exclusively in the orbit. Bahrami et al. found a *TERT* promoter mutation in 28% (26/94) of a collective of 94 extra-meningeal SFTs [31]. Although the mutation occurred in 13/31 thoracic SFTs (42%) and in 13/63 (21%) of the extra-thoracic tumors, the difference was not statistically significant (*p* = 0.248). Conclusively, the study found that *TERT* promoter mutation is significantly linked to high-risk properties (e.g., older age, larger tumor size, higher mitotic rate). A review by Liu et al., which reported both extra-meningeal and meningeal SFTs, found an overall prevalence of 24% (14/58) [30]. A large study conducted by Demicco et al. found a *TERT* promoter mutation in 29% (54/189) of tumors, including relapses and metastatic disease [52]. In this study, *TERT* promoter mutations were more common in high-risk tumors (45% 9/20), but also occurred in tumors of moderate (42%, 11/26) or low risk (21%, 14/67). Interestingly, the study reported a *TERT* promoter mutation prevalence of 2/15 (13%) of primary SFTs in the head and neck, which is similar to the prevalence in our study cohort. While these studies referred to mostly extra-meningeal SFTs, a large study from 2013 on *TERT* promoter mutations in 1515 CNS tumors found *TERT* promoter mutations in 50% (8/16) of meningeal SFTs [41]. However, the authors further differentiated a meningeal hemangiopericytoma and describe a prevalence of 11% (3/27). Thus, in conclusion, the meningeal SFTs in this study have a prevalence of 26% (11/43). Because *TERT* promoter mutations are most common in CNS tumors, some authors hypothesize that meningeal SFTs have a higher prevalence of *TERT* promoter mutations than extra-meningeal SFTs [32,53]. In contrast to this, the large series study carried out by Demicco et al. found no *TERT* promoter mutation in primary meningeal SFTs (0/14) [52]. The aforementioned studies also showed that C228T mutations are more frequent than C250T mutations [31,41]. In our study on oSFTs, the sole *TERT* promoter mutation was C250T.

OSFTs exhibit unique properties among the extra-meningeal SFT entity. While local recurrence rates are higher than in other extra-pleural or pleural locations (26% vs. 10%), the risk for metastatic disease is relatively low (2.6% vs. 5–25%), most likely due to early detection and smaller tumor size [3,16]. Hence, the orbital manifestation of SFTs manifests in younger patients (mean 42 years, in our collective mean 46 years) compared to SFTs of other compartments like the pleura (60 years) and the meningeal compartment (50 years) [3,5]. On the contrary, meningeal SFTs have a high rate of local recurrence and an overall poorer prognosis. This is reflected in a generally more aggressive histopathologic pattern with higher cellularity and higher mitotic activity and ki-67 index [5,20]. Further factors that seem to have an influence on the outcome are mitoses per HPF and the presence of tumor necrosis. These are used in a variety of risk prediction models for extra-meningeal SFTs [25,26]. Thompson et al. proposed a risk score exclusively for oSFTs, which includes tumor cellularity and cellular/nuclear polymorphism [16]. Moreover, this score accounts for the risk of local recurrence rather than distant metastasis, which is much more frequent in the treatment of orbital manifestation. Interestingly, in the present study, the metastasis risk, according to the score by Demicco et al., was only low to intermediate. Nevertheless, the two cases with intermediate metastatic risk were the cases where eye preservation could not be achieved and who harbored a STAT6-TAD fusion variant. One of them showed a *TERT* promoter mutation. This is in accordance with Bahrami et al., where the *TERT* promoter mutation only occurred in moderate- to high-risk tumors when classified according to the risk score by Demicco et al. [31]. In the present study, *TERT* promoter occurred in a case that had an unfavorable outcome, including multiple local recurrences, eye loss, and infiltrative uncontrolled local growth.

The strong association between mutations of the *TERT* promoter and malignant SFTs has previously been shown [54]. *TERT* promoter mutations are more frequent in cancers derived from tissue with low potential for self-renewal and mostly occur at an early stage of molecular dedifferentiation [40]. Acting as driver mutations, they facilitate molecular tumor progress by immortalizing the cell [35]. Mechanistically, the mutated *TERT* promoter sequence forms a de novo binding site with increased affinity for an ETS, such as GABP (see Figure 1) [39]. GABPA, which only possesses the ETS binding domain, must form a heterodimer with GABPB1 or GABPB2, that each only harbor the transactivation domain [40]. When undergoing steric DNA alterations such as chromatin looping, multiple ETS binding sites can converge, forming heterotetramers for reinforced transcription activation [40]. The induced *TERT* then joins the telomerase complex to complete the functional enzyme for telomere elongation [40]. In contrast, a study by Demicco et al. did not find a difference in telomere length when comparing SFTs with a wild-type promoter to SFTs with a mutated *TERT* promoter [52]. The authors hypothesize that there might be factors in wild-type tumors that equally lead to telomere elongation like post-transcriptional and epigenetic modification. Epigenetic alterations of the *TERT* promoter have already been described [55,56]. Although germ line variants in the *TERT* promoter have been described in other cancers, e.g., melanoma or glioblastoma, the described hotspot mutations represent somatic mutations and a germ line hot spot mutation that, to our knowledge, has never been described [35,36,37,38].

In our cohort and due to the study’s retrospective design, we only analyzed tissue retrieved during tumor surgery. Thus, we can only postulate that the C250T mutation could be a somatic mutation. As in previous studies considering SFTs in the orbital compartment, a limitation is the low number of cases, which mirrors the incidence of this rare tumor entity at this specific location. In order to increase the number of samples and draw conclusions with statistical significance, a multicenter study is actually in conception. Another limitation is the imaging availability and quality, which relied partly on scans from external facilities. This was due to the retrospective nature of the study design.

The manifestation of SFTs in the orbit is rare. Nonetheless, incidence in the early years before the recognition of NAB2::STAT6 fusion gene might be underestimated [11]; in the first two decades after the characterization of CD34 in immunohistochemistry by Westra et al. in 1994, only 80 cases of reported oSFT appeared in the English literature [11,42]. Yet, as uncovering the NAB2::STAT6 fusion gene has led to increased diagnostic sensitivity and specificity in primary SFT diagnosis, case reports in the literature have appeared to snowball [1,3,20]. Another bias of reporting might be that the improved diagnostic standard primarily translated into the field of thoracic pathology, while oSFTs are evaluated by ophthalmo-pathologists. These factors supposedly increase the count of false negative diagnoses of oSFT and can explain the relatively low numbers of prevalence in the early years. Our work, among others, highlights the importance of the diagnosis SFT in the orbit and helps to contextualize this diagnosis with former work. To our knowledge, this study is the first to evaluate the prevalence of *TERT* promoter mutations exclusively in oSFTs. Our study supports the association between *TERT* promoter mutation and unfavorable disease outcome. Further studies are needed to evaluate whether the routine analysis of *TERT* promoter mutation status in addition to the current clinical- and histopathology-based practice may improve the risk assessment for patients with oSFT.

## 5. Conclusions

SFT is a semi-malignant and heterogeneous soft tissue tumor that is becoming increasingly more well understood. To complement histomorphological analysis and to better estimate the prognosis of oSFTs, we encourage the further diagnostic use of molecular pathology. The prevalence of *TERT* promoter mutations in oSFT was 11% and occurred in a case with locally aggressive tumor growth and multiple uncontrollable, local recurrences. As previously shown for SFTs of other locations and supported by our results, we hypothesize that *TERT* promoter mutations in oSFTs might be related to unfavorable outcomes [16,52,54]. Hence, *TERT* promoter mutation analysis could complement existing and new risk stratification scores [57,58]. However, the evolution of a concept renders the interpretation of former studies more complicated. In spite of a new era of technological progress, the treatment of oSFTs remains challenging and further studies need to be undertaken in order to elucidate the role of molecular tumor biology in SFTs of the orbital compartment.

## Figures and Tables

**Table 1:** Epidemiological and clinical data at first presentation in the department of ophthalmology. (BCVA: best corrected visual acuity. IOP: intraocular pressure.)

Patient No.	Age at Initial Presentation	Gender	Symptoms	cc BCVA	IOP	Ophthalmological History
1	78	Female	Painless proptosis	0.6	17	None
2	33	Female	Painless proptosis	0.9	21	None
3	74	Female	Painful proptosis with visual alteration	0.6	NA	Mature cataract, rubeosis iridis, post ulcerative keratitis
4	42	Male	Painless swelling close to the lacrimal gland	1.25	18	None
5	38	Male	Painless proptosis	1	20	None
6	83	Male	Painless proptosis; diplopia in extreme gaze to the right	0.4	20	Cataract
7	23	Male	Painless swelling of the upper lid	1	12	None
8	78	Female	Painless swelling at the medial canthus	0.5	13	Post selective laser trabeculoplasty (SLT), post Trabectome PEX glaucoma
9	46	Male	Painless swelling of the medial upper lid	1.0	18	None

**Table 2:** Parameters and appearance of oSFT in multimodal imaging including contrast media CT and diffusion-weighted MRI imaging (DWI).

Patient N°	CT Native ″	T1 Native (SI) ″	T2 (SI) ″	CSA *	ADC ^	T1 CE	Morphology
1	n.a.	Hypointense	Intermediate	+	No reduction	Homogeneous	Vessels, well circumscribed, no compression
2	Hyperdense	Hypointense	Intermediate	+	Artifact	Homogeneous	Vessels, well circumscribed, compression signs
3	n.a.	Hypointense	Hypointense	−	-	Homogeneous	Vessels, well circumscribed, no compression
4	Hyperdense	Hypointense	Hyperintense	−	-	Homogeneous	No vessels, well circumscribed, infiltration in parasinuses
5	Hyperdense	Hypointense	Intermediate	−	Reduction	Homogeneous	Vessels, well circumscribed, compression signs
6	n.a.	Hypointense	Hypointense	−	Reduction	Inhomogeneous	Vessels, well circumscribed, no compression
7	Hyperdense	Hypointense	Intermediate	−	Reduction	Inhomogeneous	Few vessels, inhomogeneous intratumoral lesions, compression signs, suspicion of osseous infiltration
8	Hyperdense	Hypointense	Intermediate	+	Artifact	Homogeneous	Vessels, well circumscribed, no compression
9	Hyperdense	Hypointense	Intermediate	−	Reduction	Inhomogeneous	No vessels, well circumscribed, inhomogeneous, no compression

″ Qualitative visual assessment of density or signal intensity (SI) compared to surrounding soft tissue; * CSA (chemical shift artifact); ^ Reduction quantified as a mean ADC value of less than 1000 × 10^−6^ mm^2^/s; n.a. (not available).

**Table 3:** Tumor surgery included eye preserving techniques and definitive tumor resection. Definitive tumor resection included bulbar enucleation and orbital exenteration. The frequency of surgery in one patient ranged between 1 and 14 surgeries. In 7 of 9 patients, eye preservation was achieved. In one case, orbital exenteration was recommended due to aggressive local recurrence. The patient refused surgery.

Patient N°	Age at Initial Presentation	Surgical Procedure	Eye Preservation	Overall Survival (Months)	Longest Survival w/o Progress (Months)
A	78	Swinging eyelid, medial orbitotomy	Yes	42.5	-
B	33	Excisional biopsy, incomplete resection via pterional approach Anterior orbitotomy with enucleation and tumor dissection	No	91	89
C	74	Pterional craniotomy and orbitotomy, lateral orbitotomy, excisional biopsy	Yes (but recommendation for exenteration)	198	198
D	42	Lateral orbitotomy, lateral tarsal strip with symblepharolysis after cicatricial ectropion	Yes	43.5	Not available
E	39	Lateral orbitotomy, lateral tarsal strip and symblepharolysis after cicatricial ectropion	Yes	54.5	Not available
F	83	Lateral orbitotomy, lateral orbitotomy, lateral orbitotomy	Yes	82.5	36
G	23	Transcutaneous resection, lateral orbitotomy	Yes	99	68
H	78	Local excision of medial canthus, second resection of medial canthus with lateral rhinotomy and reconstruction of the medial orbital wall, symblepharolysis with amniotic membrane transplantation, navigation-device-assisted parasinus revision surgery (06/2013), tumor resection of the medial canthus with reconstruction of the medial canthus with free dermal graft, resection of an infrabulbar relapsing tumor, tumor resection, revision surgery of the orbit and parasinuses due to locally uncontrolled tumor growth, orbital exenteration and hemimaxillectomy, hemimaxillectomy due to local recurrence, repetitive tumor debulking surgery (anterior septum, nasal bridge), tumor debulking (left parasinuses and orbital bone), tumor debulking surgery (maxilla and parasinuses), tumor debulking of the parasinuses, left orofacial region and paramedian region.	No	153.5	11
I	46	Lateral orbitotomy, excisional biopsy, lateral orbitotomy	Yes	132	132

**Table 4:** Representative histopathological changes in 9 samples. Some patients had multiple resections with different specimens. We analyzed the relevant specimens with conventional histology and chose to present the most representative samples. Representative pictures of H&E staining are shown in Figure 3.

Patient N°	Diameter in mm	Necrosis	Resection Status	Number of Mitotic Figures Per mm^2^ (per 10 HPF *)	Risk for Metastasis Demicco et al. [24]	Risk for Recurrence Thompson et al. [16]
A	12	No	R0	<1, (1, 5)	Low	Low
B	39	No	R1	<1, (1)	Low	Intermediate
C	Min. 50	No	Not available	3, (13)	Intermediate	Intermediate
D	20	No	R1	<1, (3)	Low	Low
E	32	No	R1	0	Low	Intermediate
F	Min. 35	No	Not available	<1, (1)	Low	Intermediate
G	Min. 20	No	Not available	<1, (2)	Low	Low
H	Min. 180 (Pos 1–7)	No	R1	<1, 3	Intermediate	Intermediate
I	Min. 13	No	Not available	5, (18)	Low	High

* High-power field.

**Table 5:** Quantity and intensity of immunohistochemistry. Some patients had multiple resections with different specimens. We analyzed the relevant specimen with immunohistochemistry and chose the most representative samples to be depicted. Representative pictures of immunohistochemistry are shown in Figure 3.

Patient N°	CD34	STAT6	Ki-67 Index (%)	CD99
A	+++	++	3	+
B	+++	+	5	+
C	+++	+++	25	+
D	+++	+++	1	+
E	+++	+++	4	+
F	+++	+	10	++
G	−	+	30	−
H	+++	+	20	+++
I	+++	+	20	++

+ = weak positive staining in tumour cells; ++ = moderate positive staining result in tumour cells; +++ = strong positive staining result in tumour cells; − = negative staining result in tumour cells.

**Table 6:** Sanger sequencing showed one *TERT* promoter mutation (C250T) in all of the 9 tumors. All tumors were investigated for *TERT* promoter mutation. If samples from different surgeries of different time points were available, we checked selected specimens to rule out molecular tumor progress. Patient H showed a consistent C250T mutation that was already shown in specimens from the first tumor resection.

Patient N°	Eye Preservation	Local Recurrence	NAB2-STAT6 Fusion Variant	*TERT* Promoter Mutation
A	Yes	Yes	NAB2ex3::STAT6ex19	WT
B	No	Yes	NAB2ex6::STAT6ex17	WT
C	Yes (but exenteration recommended)	Yes	NAB2ex4::STAT6ex2	WT
D	Yes	No	NAB2ex3::STAT6ex19	WT
E	Yes	No	NAB2ex2::STAT6ex2	WT
F	Yes	Yes	NAB2ex6::STAT6ex17	WT
G	Yes	Yes	NAB2ex4::STAT6ex4	WT
H	No	Yes	NAB2ex3::STAT6ex19	C250T
H (+11 months)			NAB2ex3::STAT6ex19	C250T
H (+8 years, 9 months)			NAB2ex3::STAT6ex19	C250T
I	Yes	Yes	Not Available	WT

## Data Availability

No new data were created or analyzed in this study. Data are contained within the article and Supplementary Materials.

## Supplementary Materials

The following supporting information can be downloaded at: https://www.mdpi.com/article/10.3390/cimb46020095/s1.
