# Supplementary material for: Prevalence of TERT Promoter Mutations in Orbital Solitary Fibrous Tumors
Source: Curr Issues Mol Biol. 2024 Feb 10;46(2):1467–84. doi: 10.3390/cimb46020095 (PMC10887834; doi:10.3390/cimb46020095)
Supplement: Supplementary file 1 [file cimb-46-00095-s001.zip › cimb-2805920-supplementary.pdf]

Supplemental Table S1. Extended results of representative histopathological changes in 9 samples. Some patients had multiple resections with different specimen. We analysed the relevant specimen with conventional histology and chose to present the most representative samples. Representative pictures of H&E staining are shown in Fig. 3.

* HPF (high power-field)

Patient N°	Growth pattern and cellular morphology	Cellularity	Diametre (mm)	Necrosis	Vascularization	Staghorn vessels	Hyalinization	Resection status	Number of mitotic figures per mm² (*per 10 HPF**)	Risk for metastasis Demicco et al.[24]
A	Patternless pattern 50% spindled cell, 50% epitheloid cell apperance, condensed chromatin,	+++	12	No	+++	yes	-	R0	<1, *(1,5)*	Low
B	Patternless pattern spindled cell, appearance, vesicular chromatin	++	39	No	++	no	++	R1	<1, (*1*)	Low
C	Patternless pattern spindled cell, appearance, vesicular chromatin	++	Min. 50	No	+	no	+	Not available for this sample	3, (*13)*	Intermediate
D	Storiform pattern spindled cell, appearance, vesicular chromatin,	++	20	No	+++	yes	-	R1	<1, (3)	Low
E	Storiform pattern spindled cell, appearance, vesicular chromatin	+++	32	No	++	Yes (mildly)	+++	R1	0	Low
F	Patternless pattern spindled cell, appearance, condensed chromatin	+++	Min. 35	No	+	Yes (midly)	+	Not available for this sample	<1, *(1)*	Low
G	partially patternless pattern, (60%) partially storiform pattern (40%), spindled cell appearance, condensed chromatin,	+++	Min. 20	No	+++	yes	No	Not available for this sample	<1, *(2)*	Low
H	Patternless pattern, 50% spindled cell, 50% epitheloid cell appearance, condensed chromatin	++	Min. 180 (Pos 1-7)	No	+	no	+++	R1	<1, *3*	Intermediate
I	nested/insular-like growth pattern, mostly epitheloid appaerance (>90%), condensed chromatin	+	Min. 13	No	+	no	+	Not available for this sample	5, *(18)*	Low

+ = weak positive staining in tumour cells

++ = moderate positive staining result in tumour cells

+++ = strong positive staining result in tumour cells

- = negative staining result in tumour cells.

Supplemental Table S2. Extended immunohistochemistry panel: For reasons of differential diagnosis an antibody panel was administered. All specimens were positive for CD34 and STAT6.

Patient N°	CD34	Bcl2	CK MNF116	CD31	Vimentin	STAT6	Ki-67 -Index (%)	CD99	S100	p53	p16 cytoplasmatic	p16 nuclear
A	+++	+++	-	-	++	++	3	+	-	++, mutated pattern	+	+++
B	+++	++	-	-	-	+	5	+	-	++, wildtype pattern	Focally +	++
C	+++	+++	-	-	+	+++	20-25	+	-	+, wildtype pattern	++	+
D	+++	+++	-	-	+	+++	1	+	+	+, wildtype pattern	++	+
E	+++	+	-	-	+	+++	4	+	-	+, wildtype pattern	Focally +	Focally ++
F	+++	+++	+	-	+	+	6%	++	-	+, wildtype pattern	++	-
G	+++	+++	-	+	+	+++	15	+	-	+, wildtype pattern	++	++
H	+++	+++	-	Focally +	++	+	25	+	-	+, wildtype pattern	++	+++
I	+++	+++	Focally +	-	++	+	20	++	-	+, mutated pattern	++	++

+ = weak positive staining in tumour cells

++ = moderate positive staining result in tumour cells

+++ = strong positive staining result in tumour cells

- = negative staining result in tumour cells.
